# The Use of Bone Marrow Transplantation (BMT) or Hematopoietic Stem Cell Transplantation (HSCT) in Pediatric Patients Diagnosed With Ataxia-Telangiectasia: A Systematic Review

**DOI:** 10.7759/cureus.83304

**Published:** 2025-05-01

**Authors:** Saad Ali M Alqarni, Lujaine M Al Murayeh, Bayan Y Mushari, Noura H Alotaibi

**Affiliations:** 1 Pediatrics, Abha Maternity and Children Hospital, Abha, SAU

**Keywords:** ataxia-telangiectasia, immune system diseases, immunodeficiency disease, louis-bar syndrome, neurological disorder

## Abstract

Ataxia-telangiectasia (A-T) is a rare neurological disorder that leads to early death due to immunodeficiency, leukemia, and lymphoma. Given the underlying immune dysfunction and predisposition to hematologic cancers, bone marrow transplantation (BMT) or hematopoietic stem cell transplantation (HSCT) has emerged as a potential therapeutic strategy in pediatric patients with A-T. Therefore, longer follow-ups are needed to assess associated risks, side effects, procedures, and eligibility criteria. This systematic review aims to fill this gap by consolidating evidence from different parts of the world on the use of HSCT in pediatric patients diagnosed with A-T.

The study used the Preferred Reporting Items for Systematic Reviews and Meta-Analyses (PRISMA) guidelines to search five databases (PubMed, Web of Science, ScienceDirect, Google Scholar, and MEDLINE) for relevant published papers. The review covered studies on both classical and variant forms of A-T. The studies included are those with primary outcomes related to engraftment success, immunological reconstitution, survival rates, transplant-associated toxicity, infection prevalence, cancer management, and neurological progression. Only papers published in English between 2010 and 2024 were eligible for inclusion. Two experienced researchers independently assessed the retrieved papers for inclusion. A structured data collection sheet was used to retrieve relevant information from the selected articles. The risk of bias of the items included prospective and retrospective, cross-sectional, and cohort studies was assessed using the Newcastle Ottawa Quality Assessment Scale.

Eight studies were included, comprising various designs including prospective, retrospective, and population-based cohorts. Among these, three studies reported actual use of HSCT or BMT in pediatric patients with A-T, showing immune reconstitution and reduced infections, but limited impact on neurological decline. Reduced-intensity conditioning (RIC) was associated with better survival and fewer complications compared to myeloablative regimens. The remaining studies discussed HSCT theoretically or focused on supportive care, immunological profiles, cancer risk, or nutritional challenges. Overall, outcomes varied, with limited evidence supporting routine use of HSCT in A-T due to associated risks and uncertain long-term benefits.

In conclusion, HSCT shows potential in improving immune function and reducing infections in A-T patients. However, it has minimal effect on halting neurological progression. Given the risks and limited long-term data, HSCT is not currently recommended as a standard treatment for A-T.

## Introduction and background

Ataxia-telangiectasia (A-T), also called Louis-Bar syndrome, is a recessive disorder caused by primary immunodeficiency and mutation of the A-T mutated (ATM) gene [1]. A key clinical feature of A-T is progressive neurodegeneration that leads to extreme disability and telangiectasias of the sclera and skin [2]. Moreover, A-T exposes a patient to increased susceptibility to malignancies that affect 25% of patients with a median age of 12.5 [3]. Patients with A-T experience various immune system disorders, including lymphopenia and hypogammaglobulinemia, leading to immune deficiency with sinopulmonary infections and other malignancies [4]. Other clinical features reported in A-T include oral feeding problems, hypothyroidism, and gonadal failure, which cause low body weight with myopenia, altered body composition, and general growth failure [5]. Although the signs and symptoms can differ in severity, number of malignancies, and time of onset, only a small proportion of patients who retain ATM kinase activity develop milder disease [6].

There is no known cure for A-T, and management primarily focuses on addressing the various symptoms and complications [1]. Therefore, different therapies exist to address neurological, immune, cancer-related issues, and any other conditions linked to A-T. In many immunodeficiency syndromes, one of the most crucial therapies is allogeneic hematopoietic stem cell transplantation (HSCT) because it addresses the underlying problem [7]. However, the role of HSCT in A-T remains largely undetermined and continues to be a subject of industry debate. Due to the vicious and incurable nature of A-T, there have been few trials assessing the suitability of HSCT in A-T due to the high and unpredictable risk involved [8]. Given that the transplant-associated mortality can be high due to toxicities or graft-versus-host disease [9], the risk of fatal outcome in HSCT in A-T could be even larger. Nevertheless, a limited number of studies from different parts of the world have reported the suitability of HSCT in A-T.

This systematic review seeks to explore existing literature regarding the use of bone marrow transplantation (BMT) or HSCT in pediatric patients diagnosed with A-T. Currently, consensus on whether HSCT can be admissible as an official and viable option for the treatment of A-T remains elusive. Therefore, it is important to conduct a comprehensive review of existing evidence regarding the effectiveness of HSCT as a therapy for A-T. This systematic review aims to fill this gap by consolidating evidence from different parts of the world on the use of HSCT in pediatric patients diagnosed with A-T.

## Review

Materials and methods

This systematic review was conducted in accordance with the guidelines of the Preferred Reporting Items for Systematic Reviews and Meta-Analyses (PRISMA) [10]. A comprehensive search was carried out across four reputable medical databases - PubMed, Web of Science, ScienceDirect, and Google Scholar - to identify scholarly articles published between 2010 and 2024 that investigated the role of BMT in patients with A-T. A targeted combination of keywords and Boolean operators was used to capture relevant studies. Search terms included “ataxia-telangiectasia”, “Louis-Bar syndrome”, “bone marrow transplant”, “hematopoietic stem cell transplantation”, “HSCT”, and “pediatric.”

Eligibility, Data Extraction, and Management

All retrieved studies were independently screened and assessed for eligibility by the review team using predefined inclusion and exclusion criteria, which were developed collaboratively prior to the review process. Any disagreements during the study selection or data extraction phase were resolved through discussion until a unanimous consensus was reached.

Inclusion and Exclusion Criteria

This review included studies that examined the use of BMT or hematopoietic stem cell transplantation (HSCT) in pediatric patients diagnosed with A-T. Eligible studies included randomized controlled trials (RCTs), cohort studies, retrospective case series, observational studies, and clinical reports that investigated BMT/HSCT for immune correction, cancer treatment, or disease modification in A-T. Studies involving both classic and variant forms of A-T were considered.

Primary outcomes of interest included engraftment success, immune reconstitution, survival rates, transplant-related toxicity, infection frequency, malignancy management, and neurological progression. Studies were included if they compared transplant outcomes with conventional therapy or reported on standalone HSCT/BMT outcomes in A-T patients. Only articles published in English between 2010 and 2024 were considered for inclusion.

Statistical Data Analysis

The general characteristics of the included articles were described using the absolute and relative frequency of the number of studies and patients. The main indications and complications of HSCT were identified using information from the reviewed studies. The risk of bias of the items included in prospective/retrospective, cross-sectional, and cohort studies was assessed using the Newcastle Ottawa Quality Assessment Scale.

**Figure 1:**
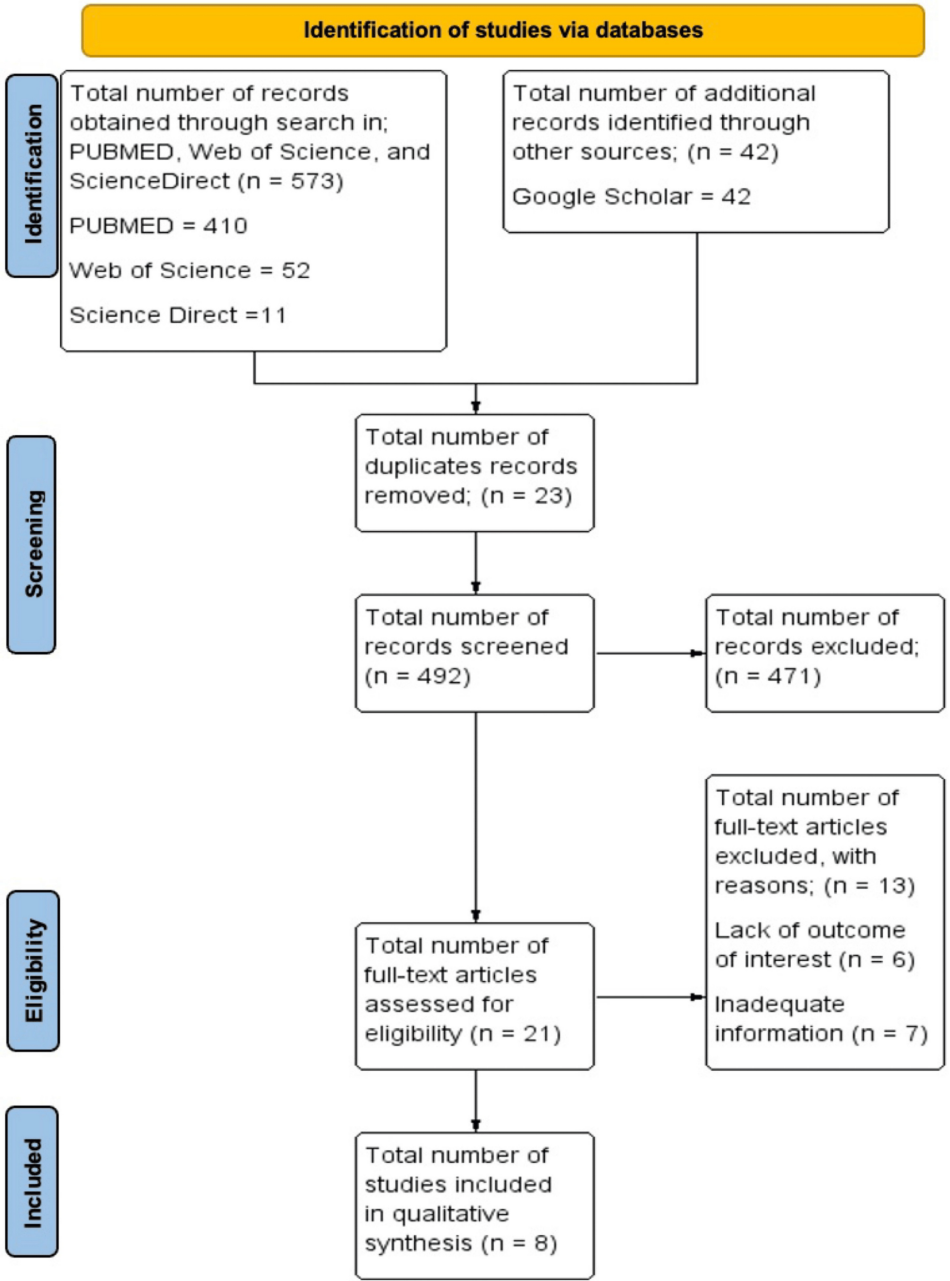
PRISMA flowchart of the study selection process PRISMA: Preferred Reporting Items for Systematic Review and Meta-Analysis

There were 515 articles found in the initial search from the various databases: PubMed - 410, Web of Science - 52, Google Scholar - 42, and ScienceDirect - 11. The studies were then screened through the above eligibility criteria, with a final number of nine studies eligible for inclusion and analysis, as illustrated in Figure 1.

Study Characteristics

The key characteristics of the reviewed studies are summarized in Table 1. All nine selected publications examined aspects of BMT or HSCT in pediatric patients with A-T, with sample sizes ranging from three to 695 participants. These studies, conducted across various countries and published in English, included both direct evaluations of HSCT outcomes and broader assessments of clinical, immunologic, or oncologic features in A-T.

**Table 1:** Overview of study designs, interventions, and outcomes in pediatrics A-T and HSCT/BMT research ALL: acute lymphoblastic leukemia; A-T: ataxia-telangiectasia; BMT: bone marrow transplantation; HSCT: hematopoietic stem cell transplantation; SCT: stem cell transplantation; SIR: standardized incidence ratio

Authors	Study type	Intervention	N	Outcome/results	Conclusion
Ussowicz et al., 2018 [2]	Prospective case series	Allogeneic stem cell transplantation (SCT) using Fanconi anemia-based conditioning regimens (GEFA02/GEFA03 protocols) in pediatric patients with A-T	Three children with classic A-T	All patients achieved stable donor T-cell engraftment and mixed donor-recipient bone marrow chimerism. Post-transplantation and immune reconstitution were observed, leading to the absence of serious infections, with only one catheter-related infection reported. Neurological deterioration occurred slowly in all patients but without immediate post-SCT toxicity. No hospitalizations were required after discharge, and no pulmonary infections developed. Growth failure persisted post-SCT.	Allogeneic SCT using modified Fanconi anemia regimens led to immune reconstitution and infection prevention in children with A-T. While it did not halt neurological progression, SCT was tolerated well and may reduce immunodeficiency-associated complications. The study highlights the need for careful patient selection and more evidence to guide SCT use in A-T.
Alyasin et al., 2019 [11]	Retrospective study	Long-term follow-up of patients diagnosed with A-T; observational focus on clinical and immunologic features. No HSCT was administered.	18	All patients presented with ataxia, and most showed signs of oculocutaneous telangiectasia, tremors, dysarthria, and immunodeficiency. Immunoglobulin deficiencies and reduced T- and B-cell counts were common. Three patients developed ALL, two of whom died. No patients received HSCT during the study period.	A-T remains a progressive, life-limiting disorder with variable immune dysfunction and high risk of malignancy. The authors emphasize the importance of early diagnosis, consistent monitoring, and supportive care but do not recommend HSCT, noting that none of the observed cases received it. Their findings support the need for improved treatment strategies and follow-up protocols.
Schoenaker et al., 2016 [12]	Retrospective study	Review of treatment approaches for acute leukemia (primarily T-ALL) in children with early-onset A-T	Pediatric A-T patients with T-ALL identified in literature	Most patients received unmodified chemotherapy, with high complete remission rates. At the time of leukemia diagnosis, many patients were not yet diagnosed with A-T. Despite high toxicity risks, reduced-intensity regimens were not always used. The authors emphasize that modified regimens should be considered but are not always applied due to delayed diagnosis. Event-free survival and long-term outcomes were variable.	Treatment of acute leukemia in children with A-T is feasible, though challenging due to high toxicity. A diagnosis of A-T is often delayed until after cancer presentation. The authors advocate for heightened clinical suspicion and early identification of A-T in young patients with leukemia, as this could allow for treatment modifications and better outcomes. HSCT was not a central intervention in this review, but the study supports multidisciplinary and tailored care.
Kilic et al., 2023 [13]	Retrospective cohort study	Clinical evaluation and immunological profiling of pediatric patients with A-T; no HSCT administered, but BMT discussed as potential future therapy	12 pediatric patients	Patients had a mean diagnosis age of 5.5 years, with consanguinity in 91.7% of cases and a family history of A-T in 58.3%. Common features included conjunctival telangiectasia (66.6%), lymphopenia (58.3%), low IgA (58.3%), IgG4 deficiency (66.7%), and impaired vaccine responses. No malignancies developed, and IVIG was started in 83.3% of patients. A-T mutations were found in 58.3%. Bone marrow transplantation was discussed as a potential option, though none of the patients received it during the study period.	The study reinforces the clinical and immunological complexity of A-T, emphasizing the need for early diagnosis, regular monitoring, and supportive care. While bone marrow transplantation was not used, it was identified as a theoretical future strategy for improving immune function and cancer prevention, though its benefit for neurological symptoms remains limited.
Slack et al., 2018 [14]	Multi-center retrospective cohort study	Hematopoietic cell transplantation (HCT) outcomes in patients with DNA repair disorders, including 8 with A-T	87 total patients (eight with A-T)	Of the 8 A-T patients, only 2 survived, both of whom received reduced-intensity conditioning (RIC) with no graft-versus-host disease (GvHD). 6 patients died, primarily from transplant-related complications such as multi-organ failure, viral activation, or PTLD. Survival was markedly higher in patients with other DNA repair disorders like LIG4 and NBS. Across the cohort, RIC had significantly better survival outcomes than myeloablative conditioning (79% vs 41%, p = 0.006).	Routine HCT is not recommended for A-T due to poor outcomes, particularly with myeloablative regimens. Although HCT can resolve immunodeficiency, the neurological decline and systemic vulnerability in A-T limit the benefit. The authors suggest that if HCT is pursued, RIC without radiotherapy is preferable, and long-term follow-up is needed to monitor for malignancies and toxicities.
Karafin et al., 2014 [15]	Retrospective cohort study	Evaluation of monoclonal and oligoclonal gammopathies in pediatric patients using serum immunofixation electrophoresis (SIFE)	695	Among 695 SIFE tests performed from 2005 to 2011, 95 (14%) revealed gammopathies in 83 patients, and 74 (11%) showed monoclonal gammopathies in 63 patients. The most common associated diagnosis was A-T (22%). No patients had multiple myeloma or Waldenström macroglobulinemia.	Monoclonal or oligoclonal gammopathies in pediatric populations have distinct patterns and implications compared to adults. In children with A-T, such findings may indicate underlying immune dysregulation rather than malignancy.
Stewart et al., 2016 [16]	Prospective observational cohort with nested case-control analysis	Longitudinal monitoring of growth parameters (weight, height, BMI) in children with A-T; evaluation of percutaneous endoscopic gastrostomy (PEG) for nutritional support	101 children with A-T	Weight, height, and BMI Z-scores were all below average, with significant declines observed over time, especially after age 8. 35% of children had weight Z-scores below -2. PEG was placed in 14% of children, and in those with longitudinal PEG data, weight trends improved (not statistically significant; p = 0.10).	Children with A-T experience progressive growth failure, particularly from age 8 onward. Early PEG feeding may help mitigate nutritional decline. The authors recommend proactive nutritional interventions, including PEG, to improve health outcomes.
Dutzmann et al., 2021 [17]	Nationwide population-based cohort study (Germany)	Linkage of genetic data with the German Childhood Cancer Registry to assess cancer incidence in children with A-T	160 pediatric patients with A-T	Among 160 children with A-T, 19 developed cancer, most commonly non-Hodgkin lymphoma (NHL) and Hodgkin lymphoma (HL). This corresponds to a 56-fold increased cancer risk compared to the general population (SIR = 56; 95% CI, 33-88). Median age at cancer diagnosis was 9.8 years. Cancer risk before age 18 was 14.3%, with high SIRs for NHL (470) and HL (215). No data on HSCT outcomes were reported, but HSCT and mosaicism were discussed as potential modifiers of risk.	Children with A-T have a substantially elevated risk of developing lymphoma and other cancers by age 18. These findings support enhanced cancer surveillance and contribute to decisions regarding early therapeutic interventions. While HSCT is not directly evaluated, its theoretical role in risk modification is acknowledged.

Risk of Bias Assessment

The quality of the included prospective studies was assessed using the Newcastle Ottawa Quality Assessment Scale (Table 2). According to the findings, two out of eight of the included studies were found to be of good quality and had a low risk of bias [15,18]. Six of the eight studies, meanwhile, showed a moderate risk of bias [11-14,16,17]. However, none of the studies had a high risk of bias. Therefore, the included studies were of moderate quality.

**Table 2:** Newcastle Ottawa Quality Assessment Scale A study was given up to one star (*) for each numbered item in the outcome and selection categories. Comparability, however, was rated as high as two stars according to the evaluation's findings (**). * demonstrates a low risk of bias in both selection and outcome. However, in terms of comparability, ** indicates a low chance of bias, whereas "-" indicates a significant risk of bias.

Author	Selection				Comparability	Outcome			Total score (1-3 high risk), (4-6 moderate risk), (7-9 low risk)
	Representativeness of the exposed cohort	Selection of the non-exposed cohort	Ascertainment of exposure	Demonstration that the outcome of interest was not present at the start of the study	Comparability of cohorts on the basis of the design or analysis	Assessment of outcome	Was the follow-up long enough for outcomes to occur	Adequacy of the follow-up of cohorts	
Ussowicz et al., 2018 [2]	*	-	*-	*	-	*	*	*	6/9
Alyasin et al., 2019 [11]	*	-	*	*	*	*	*	-	6/9
Schoenaker et al., 2016 [12]	*	-	*	*	*	*	*	-	6/9
Kilic et al., 2023 [13]	*	-	*	*	*	*	*	-	6/9
Slack et al., 2018 [14]	*	*	*	*	*	*	*	-	7/9
Karafin et al., 2014 [15]	*	-	*	*	-	*	*	-	5/9
Stewart et al., 2016 [16]	*	*	*	-	*	*	*	-	6/9
Dutzmann et al., 2021 [17]	*	*	*	*	**	*	*	*	9/9

Discussion

A-T is a rare neurological disorder that leads to early mortality due to associated immunodeficiency, leukemia, and lymphoma [1]. BMT, particularly HSCT, has emerged as a potential therapeutic option [8]. However, it requires non-myeloablative conditioning and is associated with radio-sensitivity. Therefore, longer follow-up periods are necessary to assess risks, side effects, procedures, and eligibility criteria. This systematic review aimed to evaluate the use of BMT/HSCT in pediatric patients diagnosed with A-T.

According to Ussowicz et al., children with A-T who underwent allogeneic stem cell transplantation using a modified Fanconi anemia regimen showed immune reconstitution and reduced immunodeficiency-related complications. Despite donor T-cell engraftment and the absence of serious infections, neurological deterioration persisted, indicating that HSCT does not halt disease progression. Nevertheless, HSCT was well tolerated, with no major toxicities or post-discharge hospitalizations. This underscores the need for careful patient selection and further research to optimize HSCT protocols for A-T patients [2].

Alyasin et al. emphasized that A-T remains a progressive and life-limiting condition, marked by a high risk of malignancies, especially acute lymphoblastic leukemia (ALL), and significant immunologic dysfunction. The study highlights the importance of early diagnosis, continuous monitoring, and supportive care, while stressing the need for improved treatment plans and long-term follow-up [11]. Schoenaker et al. found that treating acute leukemia in children with A-T is feasible but challenging due to the high risk of toxicity. Modified chemotherapy regimens are required, though rarely applied due to delayed A-T diagnoses. Many patients are only identified after leukemia progression. The study stresses the importance of early detection of A-T in children with leukemia to allow for treatment modifications that can minimize toxicity and improve outcomes, even though the focus was not on HSCT [12].

Kilic et al. emphasized early diagnosis, monitoring, and supportive therapy in managing A-T’s clinical and immunologic complications. Consanguinity and family history were common, with immunologic deficiencies such as lymphopenia, low IgA, and poor vaccine responses. Although intravenous immunoglobulin (IVIG) therapy was widely used, no cancers developed during the study period. BMT was not applied, and its effectiveness in addressing neurological symptoms remains unknown. The study highlights the need for further investigation into more effective treatment strategies for A-T [13].

Slack et al. reported that hematopoietic cell transplantation (HCT) in A-T carries high risks, especially with myeloablative conditioning, which is associated with increased transplant-related mortality. Only patients receiving reduced-intensity conditioning (RIC) survived without developing graft-versus-host disease (GvHD), suggesting that RIC without radiation may be a safer approach. While HCT may correct immunodeficiency, it is not routinely recommended due to persistent neurological degeneration and systemic vulnerabilities. Long-term follow-up is essential to evaluate malignancies and toxicities in transplanted patients [14].

Karafin et al. observed a higher prevalence of monoclonal and oligoclonal gammopathies in children with A-T. These were associated more with immune dysregulation than malignancy. Unlike adults, children with these gammopathies did not show links to Waldenström macroglobulinemia or multiple myeloma. These findings suggest gammopathies in pediatric A-T patients may serve as early markers of immune dysfunction rather than cancer [15].

Stewart et al. reported significant declines in height, weight, and BMI Z-scores in children with A-T, especially after age eight. Although percutaneous endoscopic gastrostomy (PEG) feeding improved weight trends, the results were not statistically significant. This suggests potential benefits of PEG, warranting further investigation. Early nutritional interventions could help prevent deterioration and improve outcomes in A-T patients [16].

Dutzmann et al. found that children with A-T had a 56-fold increased risk of developing cancer, particularly Hodgkin lymphoma (HL) and non-Hodgkin lymphoma (NHL), compared to the general population. The likelihood of developing cancer by age 18 was 14.3%, with standardized incidence ratios (SIRs) of 470 for NHL and 215 for HL. While HSCT and genetic mosaicism were considered potential modifiers of cancer risk, they were not specifically evaluated. The study emphasizes the importance of enhanced cancer surveillance and early therapeutic interventions [17].

van Os et al. reported that HSCT may improve immunologic dysfunction in A-T, with some promising results. However, due to limited clinical evidence, short follow-up durations, and potential adverse effects, routine use is not currently recommended. The study underscores the value of interdisciplinary, personalized care and advocates for further research to assess long-term outcomes of HSCT and other emerging therapies for A-T [18].

This review highlights both the potential and the limitations of BMT and HSCT in pediatric A-T patients. Limitations include short follow-up periods, small sample sizes, and inconsistent treatment protocols across studies. While HSCT may improve immune function, it does not prevent neurodegeneration, reinforcing the need for alternative treatment approaches. Regular HSCT is not recommended due to high risks, particularly with myeloablative conditioning. Early cancer detection, effective screening, and supportive nutritional care remain essential aspects of comprehensive A-T management.

## Conclusions

Currently, there is no definitive cure for A-T, and treatment is primarily symptomatic despite advances in supportive care. HSCT has emerged as a potential option but remains controversial due to its high associated mortality risk. This study finds RIC without radiation to be a safer alternative. A-T poses a significant cancer risk in children, particularly for HL and NHL, and is linked to immune dysfunction and impaired development. Future research should focus on optimizing HSCT protocols, investigating pharmaceutical approaches, enhancing cancer surveillance, and conducting long-term nutritional and developmental follow-ups.
